# Clinical characteristics and long-term outcomes of 12 children with vitamin D-dependent rickets type 1A: A retrospective study

**DOI:** 10.3389/fped.2022.1007219

**Published:** 2022-11-04

**Authors:** Yunting Lin, Zhihong Guan, Huifen Mei, Wen Zhang, Zhizi Zhou, Ling Su, Jing Cheng, Ruidan Zheng, Cuili Liang, Yanna Cai, Xi Yin, Dongyan Wu, Li Liu, Chunhua Zeng

**Affiliations:** Department of Genetics and Endocrinology, Guangzhou Women and Children's Medical Center, Guangzhou Medical University, Guangdong Provincial Clinical Research Center for Child Health, Guangzhou, China

**Keywords:** vitamin D-dependent rickets type 1A, *CYP27B1* gene, clinical characteristics, genetic spectrum, therapeutic effect

## Abstract

**Purpose:**

Vitamin D-dependent rickets type 1A (VDDR1A) is a rare autosomal recessive disorder caused by deficiency of the *CYP27B1* gene. This study aims to investigate the phenotypic and genotypic features of VDDR1A children in southern China and evaluate the long-term therapeutic effects.

**Methods:**

Twelve children from southern China with VDDR1A were enrolled in this study. Their clinical, radiological, biochemical, and molecular findings were analyzed retrospectively. The rickets severity score (RSS), biochemical parameters, and height standard deviation score (HtSDS) were used to evaluate clinical outcomes.

**Results:**

Six males and six females were included in this VDDR1A cohort. The age of onset was from 6 months to 1.8 years, and the age at diagnosis was 2.1 ± 0.8 years. The most common clinical symptoms at diagnosis were delayed walking (10/12) and severe growth retardation (9/12). HtSDS at diagnosis was negatively associated with age (*p *< 0.05). All patients presented with hypocalcemia, hypophosphatemia, increased serum alkaline phosphatase and parathyroid hormone, and high RSS at diagnosis. Two allelic variants of the *CYP27B1* gene were identified in all patients, including nine different variants, four known and five novel, with c.1319_1325dupCCCACCC(p.Phe443Profs*24) being the most frequent. All patients were treated with calcitriol and calcium after diagnosis, and all patients but one were followed-up from 6 months to 15.6 years. HtSDS, RSS, and biochemical parameters were found to be improved during the first few years of the treatment. However, only five patients had good compliance. Although RSS and biochemical parameters were significantly improved, the HtSDS change was not significant from the time of diagnosis to the last visit, and seven patients remained of a short stature (HtSDS < −2).

**Conclusion:**

Our study extends the mutational spectrum of VDDR1A and finds a hotspot variant of the *CYP27B1* gene in southern China. The results reconfirm the importance of early diagnosis and treatment compliance and reveal the challenge of height improvement in VDDR1A patients.

## Introduction

Vitamin D is essential for bone growth, calcium metabolism, and phosphate homeostasis. Nutritional deficiency of vitamin D is the most common cause of rickets, which is characterized by poor bone mineralization and, ultimately, skeletal deformities. Vitamin D-dependent rickets (VDDR) is a group of hereditary rickets with vitamin D deficiency including defects of vitamin D activation or receptors (1, 2).

VDDR type 1A (VDDR1A) is a rare autosomal recessive disorder caused by deleterious variants of the *CYP27B1* gene located on chromosome 12p13.3. The *CYP27B1* gene, comprising nine exons and eight introns, encodes a 25-hydroxyvitamin D_3_ [25(OH)D_3_] 1α-hydroxylase, which plays an important role in the biosynthesis of active vitamin D, 1,25-dihydroxyvitamin D_3_ [1,25(OH)_2_D_3_, calcitriol] (3).

The VDDR1A patients usually develop growth retardation, hypotonia, muscle weakness, motor delay, seizures, and skeletal deformities during the first 2 years of life, characterized by hypocalcemia, hypophosphatemia, elevated serum levels of alkaline phosphatase (ALP) and parathyroid hormone (PTH), normal to elevated 25-hydroxyvitamin D [25(OH)D], and decreased or inappropriately normal 1,25(OH)_2_D_3_ (4, 5). Some VDDR1A patients may present with normocalcemia or significant hypophosphatemia, leading to misdiagnosis of nutritional vitamin D deficiency rickets or hypophosphatemic rickets (6).

Clinical heterogeneity and variability have been observed in VDDR1A patients, ranging from a mild form of almost normal to a classic form of rickets and an even more severe form accompanied by dilated cardiomyopathy or green stick fracture (7).

VDDR1A was first described early in 1961 and has been reported in more than 100 patients with widely divergent ethnic backgrounds, including Turkish, Filipino, Polish, Chinese, and Canadian (8, 9). The patients with VDDR1A require lifelong treatment with calcitriol [1,25(OH)_2_D_3_] to normalize the clinical and laboratory abnormalities. However, there is limited information about large-scale cohort studies and long-term follow-up observations (1).

To date, only 90 different variants of the *CYP27B1* gene have been reported in the literature and documented in HGMD (Professional 2022.2). Among them, 81 variants are known to cause VDDR1A, and the c.1319_1325dupCCCACCC(p.Phe443Profs*24) variant is the most frequent one, whereas the c.195 + 2T > G splicing variant has been recognized as the most prevalent variant in Turkish patients (1, 4, 6, 10). It remains inconsistent whether a genotype–phenotype correlation exists in VDDR1A (1, 3, 11, 12).

In the present study, we analyzed the clinical, radiographic, biochemical, and genetic findings of 12 patients with VDDR1A from southern China to study the clinical and mutational characteristics of the patients with VDDR1A and assess the long-term therapeutic effects of current treatment.

## Materials and methods

### Patients

Twelve VDDR1A children from 12 unrelated families were enrolled in Guangzhou Women and Children's Medical Center from January 2006 to May 2022. All subjects are from southern China and are of Han ethnicity. The medical history and clinical phenotypes were collected and evaluated by specialist physicians, x-ray examinations were performed by radiologists, renal ultrasonography was performed to assess the presence of nephrocalcinosis, and biochemical parameters of serum samples were detected in the hospital's clinical laboratory center.

### Genetic analysis

Twelve VDDR1A children and their parents were subjected to genetic analysis. Genomic DNA was extracted from peripheral blood samples using DNeasy Blood and Tissue Kit (QIAGEN, Hilden, Germany). All the nine exons together with adjoining intron boundaries of the *CYP27B1* gene (NG_007076.1, NM_000785.4) were amplified using specific primers and sequenced using an ABI 3730xl DNA Analyzer (ABI, Foster City, USA). The sequencing results were analyzed using Chromas and DNAMAN software. The SNP and HGMD databases were employed to exclude the polymorphic alleles and confirm known pathogenic variants, respectively. For novel variants absent from the SNP and HGMD databases, in silico analyses with PROVEAN, SIFT, PolyPhen-2, and MutationTaster were conducted.

### Radiographs and rickets severity scores

Radiographs were routinely performed on all 12 VDDR1A patients at diagnosis and repeated at 1–3 yearly intervals after treatment. The radiographs were assessed and analyzed for RSSs independently by two researchers blinded to the patients' information. According to Thacher score methods, the wrist was scored from 0 to 4 and the knee was scored from 0 to 6; then, the scores of the wrist and the knee in each patient were added to obtain a total RSS or Thacher score (0–10), with the highest score of 10 representing the greatest severity of rickets (13, 14). The patient with only wrist or knee radiograph at diagnosis or during follow-up visit would not have RSS data. ΔRSS (RSS at the last visit minus RSS at diagnosis) was calculated and analyzed.

### Treatment and follow-up

Medical treatment records in all patients before being referred to our center, at diagnosis, and during follow-up were carefully reviewed and collected. All patients after diagnosis were treated with calcitriol and calcium. Few patients were treated with oral phosphate if they presented with moderate (0.32–0.80 mmol/L) or severe (less than 0.32 mmol/L) hypophosphatemia and significant symptoms such as muscle weakness, bone pain, and myocardial and/or respiratory compromise at diagnosis for a few days during hospitalization (15). Clinical follow-up in most patients was performed 1 month after diagnosis and subsequently at an interval of 3–6 months. The height, weight, and related biochemical parameters were measured at every visit, while renal ultrasonography was performed every 1–3 years. The height standard deviation score (HtSDS) compared with the age-specific reference value of height in normal Chinese children was calculated at diagnosis and during follow-up (16). ΔHtSDS (HtSDS at the last visit minus HtSDS at diagnosis) was calculated and analyzed.

### Statistical analysis

SPSS Statistics 17.0 software was used to calculate means and standard deviations and compare means. When comparing means, Student's *t*-test was conducted for data distributed normally, whereas the nonparametric Mann–Whitney *U* test was applied for data not. A statistically significant difference was defined by the recommended two-tailed *p*-value for a relatively small-sized cohort, *p* < 0.05. Prism Graphpad was used to draw vertical scatter plots.

## Results

### Clinical characteristics at diagnosis

Among 12 VDDR1A children, six were males and six were females, with a male-to-female ratio of 1 : 1. The age of onset ranged from 6 months to 1.8 years (1.1 ± 0.4 years), and the age at diagnosis varied from 0.9 to 3.1 years (2.1 ± 0.8 years), with an average of 1.0 ± 0.7 years from onset to diagnosis (Table 1).

**Table 1:** Clinical characteristics of 12 VDDR1A children at diagnosis.

Patient	Sex	Age (years)	Delayed walking	Short stature	Weakness	Fracture history	Seizure history	Bracelet	Rib eversion	Leg deformity	Pectus carinatum	Rachitic rosary	Scoliosis
P1	F	2.7	+	+	+	+	−	+	+	+	+	−	+
P2	F	2.0	+	+	−	+	−	+	+	+	+	+	−
P3	F	1.8	+	+	+	−	−	+	−	+	−	+	−
P4	M	2.8	+	+	−	−	−	+	+	+	−	−	−
P5	M	3.1	+	+	+	−	−	+	+	−	+	+	+
P6	M	1.2	+	+	+	−	−	+	−	−	−	+	−
P7	F	1.2	+	−	+	−	−	+	+	+	−	−	−
P8	M	2.7	+	+	+	+	−	+	+	+	+	+	−
P9	M	2.5	+	+	−	+	−	+	+	−	+	+	+
P10	F	2.7	−	+	−	−	−	−	+	+	−	−	−
P11	M	0.9	−	−	−	−	−	+	+	+	+	−	−
P12	F	1.5	+	−	−	−	−	+	+	+	−	−	−
Mean ± SD		2.1 ± 0.8											
Ratio			10/12	9/12	6/12	4/12	0	11/12	10/12	9/12	6/12	6/12	3/12

M, male; F, female; +, Yes; −, No; SD, standard deviation.

The most frequent symptomatic feature in 12 VDDR1A patients was delayed walking (83.3%), followed by short stature (75.0%) and weakness (50.0%). The musculoskeletal signs included bracelet (91.7%), rib eversion (83.3%), leg deformity (75.0%), pectus carinatum (50.0%), rachitic rosary (50.0%), and scoliosis (25.0%). It is worth noting that 4 of the 12 patients (33.3%) had a history of recurrent fracture, but none of them experienced hypocalcemic seizures (Table 1).

**Figure 1:**
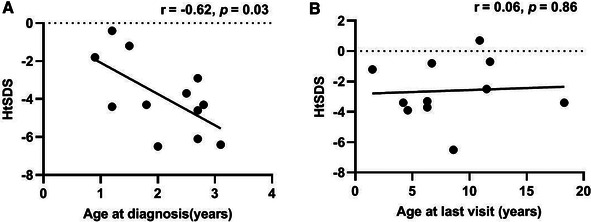
Height standard deviation score of children with VDDR1A according to age at diagnosis and at the last visit.

Among the 12 VDDR1A patients, 75.0% (9/12) underwent severe growth retardation with HtSDS below −2 of the pediatric reference range. The mean value of HtSDS in all patients was −3.8 ± 2.1 (ranging from −6.5 to −0.4) at diagnosis, and the value of HtSDS was negatively associated with age at diagnosis (*r* = −0.62, *p* < 0.05) (Figure 1A).

All 12 VDDR1A patients had hypocalcemia, hypophosphatemia, very high ALP levels, secondary hyperparathyroidism, and normal or near-normal 25(OH)D level at diagnosis (Table 2). Three patients (P1, P9, and P12) had moderate hypophosphatemia with serum phosphate 0.49–0.67 mmol/L. Serum 1,25(OH)_2_D_3_ was not available in these patients due to the technical limitations.

**Table 2:** Growth retardation and biochemical changes of 12 VDDR1A children at diagnosis and after treatment.

Patient	Age (years)	Height (SDS)	RSS	Calcium (mmol/L)	Phosphate (mmol/L)	ALP (U/L)	PTH (pmol/L)	25OHD (nmol/L)
Reference range				2.24–2.74	1.29–1.94	118–390	1.2–7.1	50–150
**P1** *								
At diagnosis	2.7	−6.1	NA*	1.38	0.49	887	96.1	46.1
After 10.4 years	13.5	−3.0	5.0	2.12	1.00	362	NA	NA
At the last visit (15.6 years)	18.3	−3.3	NA*	2.30	1.05	240	21.1	NA
**P2**								
At diagnosis	2.0	−6.5	7.5	1.68	0.92	2216	46.1	85.0
After 1.9 years	3.9	−4.9	5.0	2.43	1.39	464	2.6	NA
At the last visit (4.3 years)	6.3	−3.7	2.5	2.37	1.25	324	11.0	NA
**P3** *								
At diagnosis	1.8	−4.3	8.0	1.56	1.01	2103	24.7	104.1
After 2.7 years	4.6	−3.3	NA*	1.74	0.92	1115	23.3	NA
At the last visit (4.5 years)	6.3	−3.3	7.0	2.18	1.25	474	32.5	NA
**P4**								
At diagnosis	2.8	−4.3	9.0	1.95	0.88	1526	38.6	85.7
At the last visit (1.4 years)	4.2	−3.4	NA*	2.38	1.74	265	1.2	NA
**P5** *								
At diagnosis	3.1	−6.4	10.0	1.20	0.75	670	40.2	85.0
After 2.9 years	6.0	−4.1	6.3	2.36	1.35	156	11.6	NA
At the last visit (8.5 years)	11.5	−2.5	1.8	2.14	1.25	356	33.7	NA
**P6**								
At diagnosis	1.2	−4.4	9.0	1.54	0.94	1578	32.5	105.0
After 2.8 years	3.9	−4.1	8.8	2.09	0.87	295	10.0	NA
At the last visit (3.4 years)	4.6	−3.9	1.3	2.32	1.32	230	5.1	NA
**P7** *								
At diagnosis	1.2	−0.4	NA*	1.61	1.08	978	83.3	82.6
After 5.0 years	6.2	−0.2	5.0	2.16	1.37	185	NA	NA
At the last visit (10.6 years)	11.8	−0.7	NA*	2.07	1.17	743	47.6	NA
**P8** *								
At diagnosis	2.7	−4.6	10.0	1.50	0.72	1717	67.0	73.8
**P9** *								
At diagnosis	2.5	−3.7	9.5	1.75	0.56	1349	135.0	69.2
At the last visit (6.1 years)	8.6	−6.5	9.0	1.60	0.67	2662	91.8	NA
**P10**								
At diagnosis	2.7	−2.9	10.0	1.52	1.21	3140	48.1	47.8
After 2.7 years	5.4	−1.8	NA*	2.37	1.46	285	NA	NA
At the last visit (4 years)	6.7	−0.8	NA*	2.44	1.29	338	1.4	NA
**P11**								
At diagnosis	0.9	−1.8	NA*	1.68	1.17	1943	55.5	71.6
At the last visit (6 months)	1.5	−1.2	NA*	2.51	1.68	384	0.52	NA
**P12** *								
At diagnosis	1.5	−1.2	7.8	1.50	0.67	1446	26.7	69.8
After 6.5 years	7.9	0.0	4.5	2.42	2.12	301	NA	NA
At the last visit (9.4 years)	10.9	0.7	0.8	1.78	1.54	745	29.3	NA
Mean ± SD								
At diagnosis	2.1 ± 0.8	−3.8 ± 2.1	9.0 ± 1.0	1.57 ± 0.19	0.87 ± 0.23	1629 ± 673	57.8 ± 32.7	77.1 ± 18.4
At the last visit	8.2 ± 4.7	−2.6 ± 2.0	3.7 ± 3.4	2.19 ± 0.28	1.29 ± 0.30	615 ± 703	25.0 ± 27.3	NA

* , Poor compliance; SDS, standard deviation score; ALP, alkaline phosphatase; PTH, parathyroid hormone, 25OHD, 25-hydroxyvitamin D; SD, standard deviation; NA, not available; NA*, the patient with only wrist or knee x-ray would not have data of RSS.

**Figure 2:**
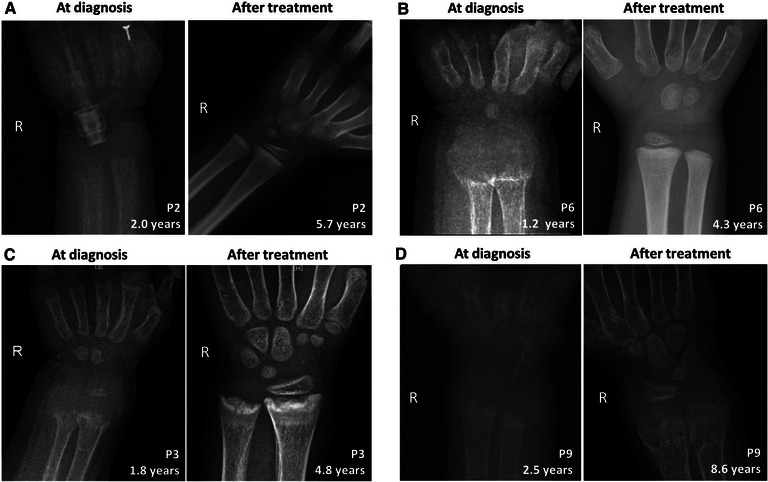
Comparison of skeletal x-ray images of four VDDR1A children at diagnosis and after treatment. In each panel, the left is the skeletal x-ray image at diagnosis, while the right is the image after treatment. P2 and P6 showed good compliance, while P3 and P9 showed poor compliance. (**A**) P2, (**B**) P6, (**C**) P3, and (**D**) P9.

The knee and/or wrist x-rays were performed in all VDDR1A patients at diagnosis. The radiographs in these patients presented with typical rachitic signs, characterized by a general decrease in bone density, widened metaphyses, and fuzzy alterations in the zone of provisional calcification in the growth plate, as shown in Figure 2. The RSS was assessed for nineVDDR1A patients with a mean value of 9.0 ± 1.0 at diagnosis (Table 2).

Renal ultrasonography was conducted in all VDDR1A patients at diagnosis. No nephrocalcinosis was revealed, and all patients had normal kidney echogenicity.

### Mutational spectrum

**Figure 3:**
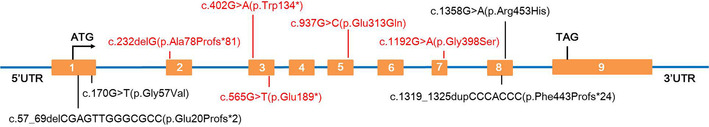
Schematic presentation of the *CYP27B1* gene with the localization of the nine different variants found in this study. Red color highlights the novel variants, while black color stands for known pathogenic variants.

Nine different variants, including four known and five novel variants, of the *CYP27B1* gene were identified (Table 3 and Figure 3). Among them, six (66.7%, 6/9) were single-base replacements, two (22.2%, 2/9) were small deletions, and one (11.1%, 1/9) was a small insertion according to the nucleotide change. When analyzing the amino acid sequence change, four (44.4%, 4/9) were missense that replaced one codon, two (22.2%, 2/9) introduced a premature stop codon, resulting in a truncated protein as nonsense, and three (33.3%, 3/9) led to a frameshift effect that altered the following sequence (Table 3).

**Table 3:** Deleterious *CYP27B1* variants identified in 12 VDDR1A children.

Patient	Exon	Nucleotide		Amino acid		Allele	Inheritance	Reported previously?
		Change	Type	Change	Type			
P1	5	c.937G > C	Replacement	p.Glu313Gln	Missense	Het	Paternal	Novel 1
	8	c.1319_1325dupCCCACCC	Small insertion	p.Phe443Profs*24	Frameshift	Het	Maternal	Known 1
P2	2	c.232delG	Small deletion	p.Ala78Profs*81	Frameshift	Hom	Parental	Novel 2
P3	8	c.1319_1325dupCCCACCC	Small insertion	p.Phe443Profs*24	Frameshift	Hom	Parental	Known 1
P4	1	c.57_69delCGAGTTGGGCGCC	Small deletion	p.Glu20Profs*2	Frameshift	Het	Paternal	Known 2
	8	c.1319_1325dupCCCACCC	Small insertion	p.Phe443Profs*24	Frameshift	Het	Maternal	Known 1
P5	1	c.57_69delCGAGTTGGGCGCC	Small deletion	p.Glu20Profs*2	Frameshift	Het	Maternal	Known 2
	3	c.565G > T	Replacement	p.Glu189*	Nonsense	Het	*De novo*	Novel 3
P6	8	c.1319_1325dupCCCACCC	Small insertion	p.Phe443Profs*24	Frameshift	Hom	Parental	Known 1
P7	8	c.1319_1325dupCCCACCC	Small insertion	p.Phe443Profs*24	Frameshift	Hom	Parental	Known 1
P8	8	c.1358G > A	Replacement	p.Arg453His	Missense	Hom	Parental	Known 3
P9	5	c.937G > C	Replacement	p.Glu313Gln	Missense	Het	Maternal	Novel 1
	7	c.1192G > A	Replacement	p.Gly398Ser	Missense	Het	Paternal	Novel 4
P10	1	c.170G > T	Replacement	p.Gly57Val	Missense	Het	Paternal	Known 4
	8	c.1319_1325dupCCCACCC	Small insertion	p.Phe443Profs*24	Frameshift	Het	Maternal	Known 1
P11	3	c.402G > A	Replacement	p.Trp134*	Nonsense	Het	Paternal	Novel 5
	8	c.1319_1325dupCCCACCC	Small insertion	p.Phe443Profs*24	Frameshift	Het	Maternal	Known 1
P12	8	c.1319_1325dupCCCACCC	Small insertion	p.Phe443Profs*24	Frameshift	Het	Paternal	Known 1
	8	c.1358G > A	Replacement	p.Arg453His	Missense	Het	Maternal	Known 3

Het, heterozygous; Hom, homozygous.

Of the 12 VDDR1A children, seven (58.3%, 7/12) carried a compound heterozygous variant and five (41.7%, 5/12) had a homozygous variant. Noticeably, the c.1319_1325dupCCCACCC(p.Phe443Profs*24) variant was found in 8 patients (66.7%, 8/12) and 11 alleles (45.8%, 11/24). Moreover, both the two pathogenic *CYP27B1* variants in most cases (91.7%, 11/12) were transmitted from parents, except for P5 with a maternal c.57_69delCGAGTTGGGCGCC(p.Glu20Profs*2) variant and a *de novo* c.565G > T(p.Glu189*) variant absent in his parents (Table 3).

Three patients (P3, P6, and P7) with the homozygous c.1319_1325dupCCCACCC(p.Phe443Profs*24) variant had similar clinical and biochemical characteristics to other 9 VDDR1A patients at diagnosis. No genotype–phenotype correlations were identified in these VDDR1A patients.

### Treatment and follow-up

Among the 12 VDDR1A patients, 8 had documented pretreatment with a physiological dose of vitamin D for a few weeks or a few months before being referred to our center. All 12 patients started treatment with calcitriol at a dose of 0.25–0.5 μg/day and calcium at a dose of 500–1000 mg/day after diagnosis. Subsequently, the calcitriol dose was titrated based on laboratory results to achieve normocalcemia and PTH levels within normal limits. Three patients (P1, P9, and P12) with moderate hypophosphatemia presented significant muscle weakness at diagnosis. Among them, two patients (P1 and P12) received phosphate for a few days during hospitalization (15), whereas another patient (P9) was initially misdiagnosed with hypophosphatemic rickets and treated with calcitriol and phosphate during hospitalization. He was lost to follow-up until he revisited our center when he was 8 years old.

Among these 12 patients, 10 patients except P8 and P9 kept regular clinic visits with good compliance during the first few years after treatment. Then, five of them (P1, P3, P5, P7, and P12) had clinic follow-ups but took medicine irregularly, particularly over 5 years after diagnosis. Patient P9 discontinued treatment and stopped clinic visits a few months after diagnosis, and P8 was lost to follow-up since diagnosis. P9 revisited the clinic 6 years after diagnosis, presenting with severe skeletal deformities and loss of the ability to stand and walk independently.

**Figure 4:**
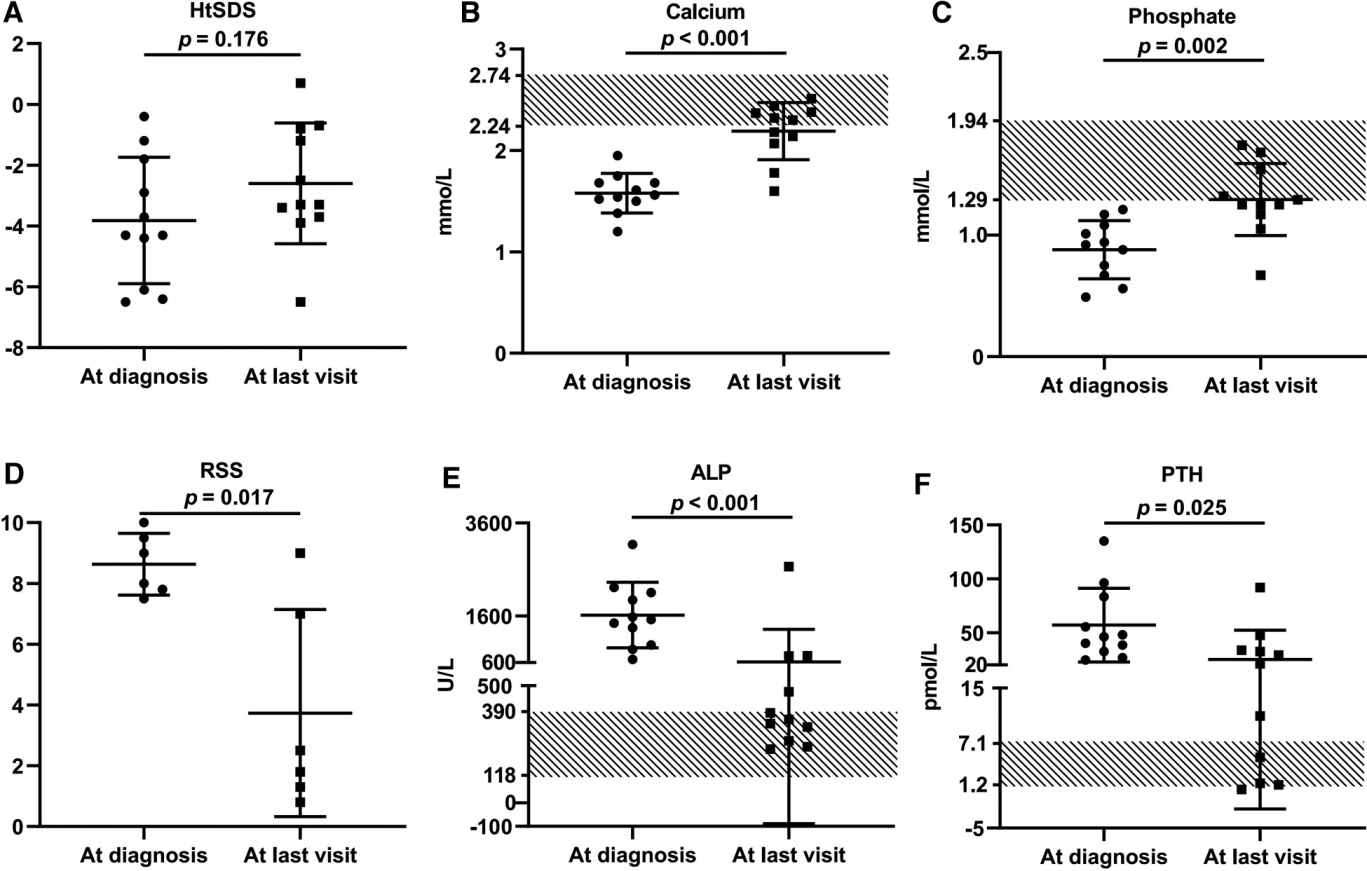
Comparison of growth retardation and biochemical changes of 11 VDDR1A children at diagnosis and after treatment. P8 was excluded from this comparison because he was lost in follow-up. (**A**) HtSDS, (**B**) calcium, (**C**) phosphate, (**D**) comparison of RSS of six VDDR1A children (P2, P3, P5, P6, P9, P12), (**E**) ALP, (**F**) PTH.

These VDDR1A patients except P8 had a follow-up of 6.2 ± 4.4 years, ranging from 6 months to 15.6 years. The results of HtSDS, RSS, and biochemical parameters of the VDDR1A patients at diagnosis, during follow-up, and at the last visit are presented in Table 2 and Figure 4. All but P9 (90.9%, 10/11) showed a catch-up growth during the first few years after treatment; only three patients (P2, P5, and P10) maintained a catch-up growth trend at the last visit. The mean HtSDS was increased from −3.8 ± 2.1 at diagnosis to −2.6 ± 2.0 at the last visit, but the change was not significant (*p* > 0.05, Figure 4A). Seven patients (63.6%, 7/11) remained of a short stature with HtSDS below −2 at the last visit (Table 2). Different from the finding at diagnosis, HtSDS at the last visit was not associated with age (*r* = 0.06, *p* = 0.86) (Figure 1B).

**Figure 5:**
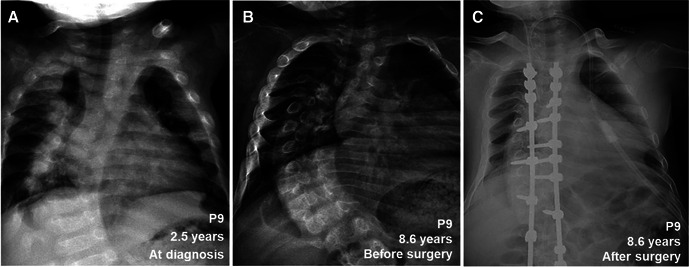
Comparison of skeletal x-ray images of VDDR1A patient P9 at diagnosis and after treatment. (**A**) At diagnosis, (**B**) after treatment was discontinued, (**C**) after surgery.

**Figure 6:**
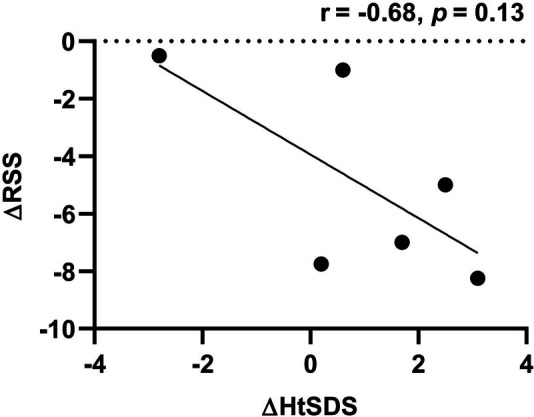
Correlation between HtSDS and RSS changes. ΔRSS was calculated by RSS at the last visit minus RSS at diagnosis. ΔHtSDS was calculated by HtSDS at the last visit minus HtSDS at diagnosis.

During follow-up, the wrist and knee radiographic findings have shown noticeable improvement in all VDDR1A patients except P8 (lost to follow-up) and P9 (no treatment). As shown in Figure 2, the patients with good compliance (P2 and P6, Figures 2A,B) presented with better skeletal improvement, whereas the other patients with poor compliance (P3 and P9, Figures 2C,D) had decreased bone density and poor calcification in the growth plate, particularly in P9. P9 received surgery due to severe scoliosis 6 years after diagnosis (Figure 5). Six VDDR1A patients had RSS at diagnosis and at the last visit. The mean value of RSS in six VDDR1A patients was 3.7 ± 3.4 at the last visit, significantly lower than 8.6 ± 1.0 RSS at diagnosis, reflecting skeletal improvement (*p* < 0.05, Figure 4D). Moreover, ΔRSS was negatively associated with ΔHtSDS (Figure 6).

Eleven patients except P8 (lost follow-up) received renal ultrasonography during follow-up, and none of them showed nephrocalcinosis. Only P12 had enhanced kidney echogenicity, while the kidney echogenicity of the other 10 patients remained normal.

With the treatment of calcitriol and calcium, the biochemical parameters (calcium, phosphate, ALP, and PTH) improved in all patients except P8 and P9 (Table 2 and Figure 4). Serum calcium and phosphate at the last visit were significantly higher than those at diagnosis (*p* < 0.001, *p* < 0.01), whereas serum ALP and PTH were significantly decreased at the last visit (*p* < 0.001, *p* < 0.05). P9 exhibited high levels of RSS, ALP, and PTH at the last visit after a lack of treatment for 6 years (Table 2 and Figure 4).

## Discussion

VDDR1A is a rare hereditary hypocalcemic rickets, and physicians in China seldom hear of this disease. To date, fewer than 30 Chinese VDDR1A patients have been reported (9, 12, 17–19). To provide a better understanding of clinical and genetic features and long-term outcomes of VDDR1A patients in southern China, we enrolled a cohort of 12 VDDR1A children.

Most cases in our cohort manifested typical clinical symptoms with delayed walking, short stature, and skeletal deformities, radiological characteristics of rachitic changes, and biochemical features of hypocalcemia, hypophosphatemia, elevated ALP, and hyperparathyroidism with onset age from 6 months to 1.8 years (4, 5). Consistent with the findings of Edouard et al., HtSDS of our VDDR1A patients was negatively associated with the age at diagnosis, indicating that older children may have more severe growth retardation (20). Early diagnosis along with long-term calcitriol treatment is critical for stature growth.

Young VDDR1A children may develop seizures due to hypocalcemia, which was reported in 11.3% (17/151) VDDR1A patients in a non-Chinese population (5). Dursun et al. reported that only one of 11 VDDR1A Turkish patients had hypocalcemic seizures (6). Similarly, Edouard et al. reported that only 4 of 21 VDDR1A Canadian patients presented with hypocalcemic seizures (20). Despite hypocalcemia in all patients, none in our cohort was symptomatic before 6 months of age, and none presented with seizure or tetany before and after diagnosis. The results highlighted the fact that the diagnosis of VDDR1A should not be excluded in children without a history of seizure or tetany.

Due to 1α-hydroxylase deficiency caused by the *CYP27B1* gene variant, VDDR1A patients are treated with calcitriol and calcium to correct the biochemical and skeletal abnormalities. All patients in this cohort were treated with calcitriol and calcium immediately after diagnosis and were well tolerated. However, with follow-up from 6 months to 15.6 years, only three patients (P2, P5, and P10) maintained a catch-up growth trend at the last visit, and the improvement of HtSDS from diagnosis to the last visit was not significant (*p* > 0.05), and seven patients (63.6%, 7/11) remained of a short stature with HtSDS below −2 at the last visit. As shown in Table 2, most cases presented with growth and motor developmental improvement with a good response to the treatment during the first few years, and biochemical parameters became normal or close to normal. After reviewing the medical records of all 12 patients and talking with the patients and their parents, we found that medicine compliance was the most important reason for treatment failure in about half of the patients. Poor compliance in these patients was associated with economic reasons, social problems, lack of medical education, and psychiatric problems during puberty, which alerts us of future work.

There were some case reports of nephrocalcinosis or corneal calcium deposits in VDDR1A patients after long-term calcitriol treatment (20). In this cohort study, renal ultrasonography was conducted in all VDDR1A patients at diagnosis and repeated in most of them during follow-up. No nephrocalcinosis was found in our patients. Further observation is needed.

All of our VDDR1A patients were proven genetically. Four known and five novel variants of the *CYP27B1* gene, totaling nine different variants, were found in our study. Of the five novel variants, four were point mutations and one was a small deletion, resulting in two missense, two nonsense, and one frameshift change in the amino acid sequence, which expands the mutational spectrum of this disease. Among the four known variants, the c.1319_1325dupCCCACCC(p.Phe443Profs*24) variant was found in eight patients (66.7%, 8/12) as a hotspot, which fits well with the previous studies based on different regions and populations (1, 6, 20–24).

## Conclusion

Our study reports 12 southern Chinese patients with VDDR1A. It describes their phenotypic and genotypic characteristics and shares our experience with the follow-up and therapeutic effects, which enriches the patient resources and clinical data. Our study also finds five novel variants of the *CYP27B1* gene and confirms that the c.1319_1325dupCCCACCC(p.Phe443Profs*24) variant is the most common variant to cause VDDR1A. The results show the importance of early diagnosis and good compliance and reveal the challenge of height improvement in VDDR1A patients.

## Data Availability

The raw data supporting the conclusions of this article will be made available by the authors, without undue reservation.
